# Status of perceived social support and its associated factors among inmate prisoners in Northwest Amhara, Ethiopia

**DOI:** 10.1186/s13104-019-4696-z

**Published:** 2019-10-17

**Authors:** Abel Fekadu Dadi, Berihun Assefa Dachew, Amare Tariku, Yohannes Ayanaw Habitu, Getu Debalkie Demissie

**Affiliations:** 1 Department of Epidemiology and Biostatistics, College of Medicine and Health Science, University of Gondar, Gondar, Ethiopia; 2 Department of Human Nutrition, College of Medicine and Health Science, University of Gondar, Gondar, Ethiopia; 3 School of Public Health, College of Medicine and Public Health, Flinders University, Adelaide, Australia; 4 Department of Reproductive Health, College of Medicine and Health Science, University of Gondar, Gondar, Ethiopia; 5 Department of Health Promotion and Behavioral Health Sciences, College of Medicine and Health Science, University of Gondar, Gondar, Ethiopia; 6 Institute for Social Science Research (ISSR), The University of Queensland, Brisbane, Australia

**Keywords:** Social support, Prisoners, Crosses-sectional study

## Abstract

**Objective:**

The objective of this study was to determine the level of social support and associated factors in selected prison institutions in Amhara region, Ethiopia.

**Result:**

Prisoners that had good social support from their family, friends, and significant others were 64.7%, (95% CI 60.9%, 68.4%). The odds of social support was higher among those educated and rural prisoners. However, it was found to be lower among non-Orthodox Christian prisoners and prisoners who were discriminated. Social support is buffering tool for social difficulties and hardships faced by prisoners while they are in prison and very helpful to reduce mental health morbidities and their consequences, hence should be strengthened.

## Introduction

Nearly 10 million people are in prisons worldwide this day and the number has been alarmingly increasing in most countries. The United State of America, China and Russia are the countries with the highest number of prisoners among developed countries while South Africa has the highest number of prisoners in Africa [1].

Prisoners shoulder a substantial burden of physical and psychiatric disorders compared to the general population. For example, the rate of depression among male and female prisoners in the United States is estimated to be 40% and 14% respectively, which is twice of the general population [2, 3]. The problem also exist in low-income countries including Ethiopia [4–7]. Many of these disorders might present before admission to prison and further exacerbated by the stress of the imprisonment. However, mental disorders might also develop during imprisonment because of prevailing conditions possibly due to torture or other human rights violations [8, 9].

Social support is critical during their stay in the prison and post-release period to ease the transition, avoid recidivism and increase the likelihood of engaging in health risk practice without being affected with any psychological morbidities [10].

A number of studies so far showed social support as having a direct relationship with mental and physical well-being [5, 11–13]. Studies conducted on the general population have also found that good social support is associated with postive mental and physical well-being [12]. For prisoners, social support might be required in order to prevent their guilty and homelessness feeling and maintain their physical as well as mental wellbeing [14]. Findings also revealed that different factors (sex, religious and education level) affect perceived social support of prison inmates [15–17].

Research that focused on determining the status of social support in prison population especially in Ethiopia is very nil. Prisoned population are more at risk of common mental disorder than the general population and because of the nature of reasons of imprisonment it could be very difficult for prisoners to maintain a support that they have been got before. So, the knowledge of how prisoners are supported and what are the factors affecting the level of their social support is very important to help organizations working on the mental wellbeing of prisoners. Therefore, the aim of this study was to assess the level of perceived social support and its predictors in selected correctional institutions in the northwest of Amhara region, Ethiopia.

## Main text

### Methods

#### Study design and setting

The institution based cross-sectional study was conducted to determine the status of social support and its associated factors among prison inmates found in prisons of North West Amhara regional state. Amhara regional state is among one of the 11 regions in Ethiopia with a total population of 19,602,512. There are 30 correctional institutions in the region while 10 of them are found in the North West part. The numbers of prisoners found in 30 correctional institutions in the region were 22,590 while 7564 prison inmates were imprisoned in correctional institutions found in the Northwest of the region.

#### Sample size determination and sampling procedure

All prisoners found in selected prisons of the North West Amhara regional state were the study populations. Those prisoners who were seriously ill and unable to communicate were excluded from the study. The minimum sample size (n) was computed by single population proportion formula [n = [(Z_a/2_)^2^*P (1 − P)]/d^2^] by assuming 95% confidence level of Z_a/2_ = 1.96, margin of error 5%, proportion (p) of 50% and the final sample size was estimated to be 662. A 1.5 design effect was used by considering the multistage sampling technique and assuming that there was no as such big variations among the prisons included in the study.

Multi-stage sampling technique was employed to select the study participants. Three correctional institutions: Bahir Dar, DebreTabor, and Gondar were randomly selected from 10 found in the Northwest Amhara Regional state. The prisoner list in every correctional institution was used as a sampling frame to randomly and proportionally select prisoners from each prison.

#### Data collection and data quality control

Data were collected by using structured interview administered questionnaire and the data collection method was through face to face interview with prisoners. It had four parts. The first part contains socio-demographic characteristics of the prisoners. The second part contained measurement tools used to assess prisoners common mental morbidity (depression, psychological distress, and anxiety). The Generalized Anxiety Disorder 7-item (GAD-7) [scale ranging from zero (not at all) to three (nearly every day)] was used to measure anxiety [18].

The Patient Health Questionnaire (PHQ-9) which contained nine questions each measuring a problem that the prisoners bothered in the last 15 days were used to measure depression and its scale measurement ranges from zero (not at all) to three (nearly every day) [19]. The Kessler Psychological Distress Scale (K10) with five-level response was used to measure psychological distress [20]. Multidimensional scale of perceived social support (MSPSS) was used to measure the level of prisoner’s social support, the main outcome variable. The tool contains 12 questions that used to assess social support that a prisoner got from his family, friends and significant others. Each item is scored from 1 (very strongly disagree to 7 (very strongly agree) [21].

Receiver operating characteristics (ROC) curve analysis was done by STATA version 12 software in order to determine a cut of the point with high sensitivity and specificity. An individual is considered as having good social support if he/she has a score above the cut-off value, which is 39. The internal consistency of the outcome variable, Multidimensional scale of perceived social support (MSPSS), was checked by conducting a reliability test (Cronbach’s Alpha: 0.952).

The third part of the questionnaire was asked to assess behavioral factors, which includes the history of substance use (like Alcohol use, Chat chewing, cigarette smoking, Shisha) of the prisoner. The last (fourth) part assessed prisoners Socio-economic and environmental factors.

Eight Bachelor of Science (B.Sc.) holder data collectors were recruited, trained, and collected the data. The tool was pretested. The collected data were reviewed and checked for completeness and consistency before data entry.

#### Data processing and analysis

Data were checked and entered by using Epi Info version 7 and imported to SPSS version 20 for further cleaning and univariate analysis. The cleaned data were exported to STATA version 12 for further analysis. Mixed effect logistic regression model was fitted to avoid the clustering effect within the prisons. Prisons effect means prisoners selected from the same correctional institution would share common environmental and prison related factors. Adjusted odds ratio (AOR) with its 95% confidence interval was used to declare statistical significance between social support and associated factors.

### Result

#### Socio-demographic characteristics of the prisoner

Data was collected from 649 respondents with a response rate of 98%. The median inter quartile range age (IQR) of the prisoners was 28 years [24–35] and the median year they would stay in the correctional institution (IQR) for a prisoner was 10 years [3, 12]. Five hundred eighty-three (89.8%) were male while 434 (66.9%) of the prisoners were from urban part of the country. (Table 1).

**Table 1:** Socio-demographic characteristics of prisoners imprisoned in selected correctional institution of Northwest Amhara, Ethiopia

Explanatory variables	Frequency (%)
Sex	
Male	583 (89.8)
Female	66 (10.2)
Residence	
Urban	434 (66.9)
Rural	215 (33.1)
Religion	
Orthodox Christian	584 (90)
Others (Muslim, Catholic and Protestant)	65 (10)
Marital status	
Single	306 (47.1)
Married	228 (35.1)
Not live with their partners	115 (17.7)
Educational status	
Not read and write	108 (16.6)
Read and write	97 (14.9)
1–8 class complete	129 (19.9)
9–12 class complete	206 (31.9)
Certificate and above	109 (16.8)

#### In prison related characteristics

One hundred thirty-eight (21.3%) prisoners were life-sentenced prisoners, 308 prisoners conduct religious practice usually while more than half (59.9%) of the prisoners participate in income generating activities in the correctional institutions. Three hundred thirteen (48.2%) prisoners have not recognised the reason for their imprisonment and majority (85.8%) of the prisoners were not agree on the number of years they have to penalise (Table 2).

**Table 2:** In prisoner related characteristics of prisoners imprisoned in selected correctional institution of Northwest Amhara, Ethiopia

Explanatory variables	Frequency (%)
Type of prisoner	
Life sentenced prisoner	138 (21.3)
Other than life Sentenced prisoner	511 (78.7)
Frequency of conduct religious practice	
Always	308 (47.5)
Sometimes	229 (35.3)
Never	112 (17.3)
Participate in income generating	
Yes	389 (59.9)
No	260 (40.1)
Did you have a job before you become prisoner	
Yes	467 (72.0)
No	182 (28.0)
Did you felt happy with your life until you become prisoner	
Yes	567 (87.4)
No	82 (12.6)
Do you have a friend in the prison	
Yes	407 (62.7)
No	242 (37.3)
Had you been discriminated because of your imprisonment	
Yes	283 (43.6)
No	365 (56.4)
How often you feel guilty	
Always	354 (54.5)
Sometimes	105 (16.2)
Never	190 (29.3)
Perceived magnitude of mistake committed	
Hard	304 (46.8)
Medium	152 (23.4)
Low	193 (29.7)
Did you believe on the crime you have committed	
Yes	267 (41.1)
No	313 (48.2)
I don’t have any idea	69 (10.6)
Is the year you penalized is in line with your mistake	
Yes	30 (4.6)
No	557 (85.8)
I don’t have any idea	62 (9.6)
Satisfaction with the care you obtain	
Satisfied	65 (10)
Medium satisfaction	580 (89.4)
Low satisfaction	4 (0.6)
Had previous psychiatric problem	
Yes	92 (14.2)
No	557 (85.8)
Family history of mental illness	
Yes	84 (12.9)
No	565 (87.1)
Did you use chat, shisha or cigarette smoking habit	
Yes	118 (18.2)
No	531 (81.8)

#### Level of social support and associated factors

In the current study, 420 (64.7%) of the prisoners had good social support (95% CI 60.9%, 68.4%) and from this 380 (905%) of them were males. From 66 female prisoners, 6.16% of them had good social support. The study identified different factors that are positively and negatively associated with prisoners social support condition.

After adjusting to different factors: prisoner’s residence before imprisonment (rural, [AOR = 0.62, 95% CI 0.41, 0.93], religion being non-orthodox[AOR = 0.48, 95% CI 0.27, 0.86] and educational status, [could be able to read and write, (AOR = 2.86, 95% CI 1.53, 5.35), completed 9–12 class (AOR = 1.95, 95% CI 1.10, 3.43) and above college level (AOR = 2.35, 95% CI 1.20, 4.59), were significantly associated.

On top of that, having friend in the correctional institution [AOR = 0.48, 95% CI 0.33, 0.70], being discriminated by the social for his/her mistake[AOR = 0.55, 95% CI 0.38, 0.80], never feeling guilty[AOR = 0.60, 95% CI 0.39, 0.91] and impossibilities to be back to the previous state [AOR = 0.62, 95% CI0.42, 0.91] were additional factors that affected the level of the prisoners social support (Table 3).

**Table 3:** Bivariate and multivariable logistic regression model fitted to identify factors associated with prisoner’s social support among prisoners imprisoned in selected correctional institution of Northwest Amhara, Ethiopia

Explanatory variables	Social support		COR_95% CI_	AOR_95% CI_
	Good	Poor		
Residence				
Urban	301	133	1	1
Rural	119	96	0.55 (0.39, 0.77)	0.62 (0.41, 0.93)*
Religion				
Orthodox	384	200	1	1
Others (Catholic, Muslim, Protestant)	36	29	0.65 (0.38, 1.09)	0.48 (0.27, 0.86)*
Marital status of the prisoner				
Single	211	95	1	
Married	143	85	0.75 (0.53, 1.09)	
Separated	66	49	0.60 (0.39, 0.95)	
Educational status of the prisoner				
Not read and write	50	58	1	1
Read and write	68	29	2.70 (1.52, 4.82)	2.86 (1.53, 5.35)*
1–8 class	76	53	1.66 (0.99, 2.79)	1.51 (0.84, 2.70)
9–12 class	145	61	2.69 (1.65, 4.39)	1.95 (1.10, 3.43)*
College and above	81	28	3.20 (1.78, 5.75)	2.35 (1.20, 4.59)*
Did you felt happy with your life until you become prisoner				
Yes	377	190	1	
No	43	39	0.54 (0.34, 0.87)	
Do you have friend in this prison				
Yes	292	115	1	1
No	128	114	0.45 (0.32, 0.63)	0.48 (0.33, 0.70)**
Have you been discriminated because of crime you have committed				
Yes	162	121	0.54 (0.39, 0.75)	0.55 (0.38, 0.80)**
No	258	108	1	1
How often you feel guilty				
Always	236	118	1	1
Sometimes	73	32	1.08 (0.67, 1.74)	0.91 (0.54, 1.55)
Never	111	79	0.71 (0.49, 1.02)	0.60 (0.39, 0.91)*
Previous psychiatric problem				
Yes	50	42	0.54 (0.34, 0.86)	
No	370	187	1	
Family history of mental illness				
Yes	49	35	0.66 (0.41, 1.07)	
No	371	194	1	
Is there any impossibilities that prevent you to resettle to the previous state				
Yes	124	89	0.57 (0.40, 0.82)	0.62 (0.42, 0.91)*
No	296	140	1	1
Psychological distress				
Distressed	340	201	1	
Not distressed	80	28	1.71 (1.07, 2.75)	
Depression				
Depressed	169	115	1	
Not depressed	251	114	1.64 (1.17, 2.30)	

Significance (P-value) ** 0.01, * 0.05

### Discussion

Nearly two out of three inmate prisoners reported that they had got social support from their friends and families. This finding showed a low level of social support as compared to the expected level that this neglected people should have. Though the social support measurement tool had a wide dimension, still it was used to assess a support that a prisoner got from his/her family, friends in the prison and other near relatives being in that prison.

This study revealed that educational status was significantly associated with prisoners’ social support. Prisoners who could read and write and more educated had significantly better in social support. This might happen because of persons with higher education level gave social value for his relatives. Other study also revealed that prisoners whose education level is beyond high school perceived that as having social support from his nearby persons [17].

The religion of the prisoner was also significantly affected the level of social support. Compared to other religious followers, orthodox religious followers were 52% higher to had good social support. A study conducted in Mississippi, prisoners who participated in Christians’ religious epiphanies got social support through engagement with similar faith followers. The epiphany participation helped to develop social support networks [22]. Generally religious participation can contribute for social support as there is shared value among every member of the group [23].

This study also showed that lack of friends in prison increased the odds of poor social support. Prisoners who did not make any friend in the correction center had a significantly low level of social support. This might happen because of the fact that having friend would give a chance for the prisoner to get relatively better support. A nearby friend would share each other happiness and sorrow that he/she felt in the correctional center. Findings showed a positive correlation between social support from friends (both inside and outside of prison) [17].

In this study, one of the factors that significantly affected the level of prisoner social support was a residence. Prisoners from rural part had low social support compared to that of urban. This might happen because of the fact that relatives from the rural part would take a long distance to reach the prisoner. This would cost them money and longtime which would be difficult for them to afford. On top of that, the number and the quality of friends that the prisoner had during his life period might be also different for prisoners from urban and rural.

The other variables that had a significant association with social support were discrimination condition, feeling guilty about the crime, and the impossibility for the prisoner to resettle to their previous state. Accordingly, the odd of having social support was 45%, 40% and 38% times lower if the prisoner was discriminated by his/her socials, felt guilty about the crime he/she committed and if it is impossible for the prisoner to resettle to his/her previous state respectively. There is a study which showed that inmates may experience greater isolation and a sense of feeling forgotten by their families, communities, and society, resulting in a greater deficit of social support and a significant relationship between social support and psychological well-being, specifically with self-esteem [24].

## Limitation

The major limitation of this study was, not using qualitative method to explore more information.

## Data Availability

The data is found on the hands of first author and could be obtained upon reasonable request.

## Abbreviations

AOR: adjusted odds ratio
BSc: Bachelor of Science
COR: Crude Odds Ratio
GAD-7: Generalized Anxiety Disorder 7-item
IQR: interquartile range
MSPSS: multidimensional scale of perceived social support
PHQ-9: Patient Health Questionnaire
ROC: receiver operating characteristics
